# A Protest

**Published:** 1875-05

**Authors:** 


*A Protest Against, and Resignation of Dr. Thos. B. Gunning* from Board of Regents of the lately started Dental College, in which he dissolves all official connection with the same, and accuses the so-called Faculty of publishing a letter leading to injurious tendencies as regards the manner in which they propose to conduct their institution. Published in pamphlet form by Dr. Gunning.

Dr. Gunning makes use of the following language in regard to this point: "Admit it once, and every city, town or community that can muster a few ambitious dentists, is authorized to have its own Dental College, the requisite being only that the Faculty shall be endorsed by the clergymen, physicians and lawyers of their acquaintance."

This is certainly to the point, and the Doctor's eyes have evidently been opened, and he discovers that "all is not gold that glitters."

